# Base excision repair but not DNA double‐strand break repair is impaired in aged human adipose‐derived stem cells

**DOI:** 10.1111/acel.13062

**Published:** 2019-11-29

**Authors:** Haiping Zhang, Bailian Cai, Anke Geng, Huanyin Tang, Wenjun Zhang, Sheng Li, Ying Jiang, Rong Tan, Xiaoping Wan, Zhiyong Mao

**Affiliations:** ^1^ Clinical and Translational Research Center of Shanghai First Maternity & Infant Hospital Shanghai Key Laboratory of Signaling and Disease Research School of Life Sciences and Technology Tongji University Shanghai China; ^2^ Clinical and Translational Research Center of Shanghai First Maternity & Infant Hospital School of Medicine Tongji University Shanghai China; ^3^ Department of Plastic Surgery Changzheng Hospital Shanghai China; ^4^ Center for Molecular Medicine Xiangya Hospital Central South University Changsha China; ^5^ State Key Laboratory of Natural Medicines China Pharmaceutical University Nanjing China

**Keywords:** adipose‐derived stem cells, base excision repair, genome integrity, human aging, XRCC1

## Abstract

The decline in DNA repair capacity contributes to the age‐associated decrease in genome integrity in somatic cells of different species. However, due to the lack of clinical samples and appropriate tools for studying DNA repair, whether and how age‐associated changes in DNA repair result in a loss of genome integrity of human adult stem cells remains incompletely characterized. Here, we isolated 20 eyelid adipose‐derived stem cell (ADSC) lines from healthy individuals (young: 10 donors with ages ranging 17–25 years; old: 10 donors with ages ranging 50–59 years). Using these cell lines, we systematically compared the efficiency of base excision repair (BER) and two DNA double‐strand break (DSB) repair pathways—nonhomologous end joining (NHEJ) and homologous recombination (HR)—between the young and old groups. Surprisingly, we found that the efficiency of BER but not NHEJ or HR is impaired in aged human ADSCs, which is in contrast to previous findings that DSB repair declines with age in human fibroblasts. We also demonstrated that BER efficiency is negatively associated with tail moment, which reflects a loss of genome integrity in human ADSCs. Mechanistic studies indicated that at the protein level XRCC1, but not other BER factors, exhibited age‐associated decline. Overexpression of XRCC1 reversed the decline of BER efficiency and genome integrity, indicating that XRCC1 is a potential therapeutic target for stabilizing genomes in aged ADSCs.

## Graphical abstract

BER but not DNA DSB repair is impaired in aged adult ADSCs.

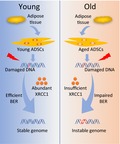

## 1 INTRODUCTION, RESULTS, AND DISCUSSION

The stem cell theory of aging assumes that age‐associated changes in adult stem cell functionality lead to a failure to preserve tissue homeostasis, eventually resulting in the onset of aging and age‐associated diseases (Goodell & Rando, 2015). As such, it is possible that stem cell therapy can benefit patients who suffer from age‐related diseases such as musculoskeletal disorders (Jo et al., 2014), neurological disorders (Lee et al., 2010), and cardiovascular diseases (De Luca et al., 2019; Patel et al., 2016). Indeed, a number of clinical trials are being conducted to treat age‐associated diseases using adult mesenchymal stem cells worldwide (Jo et al., 2014; Lee et al., 2010; Patel et al., 2016). The application of autologous stem cells in the clinic has received considerable attention as this method not only avoids ethical concerns associated with embryonic stem cells but also minimizes the risks of immune rejections. However, the extent to which the genome integrity of adult stem cells isolated from older patients influences the efficacy and safety outcomes of autologous stem cell therapy remains unclear. Accumulating evidence indicates that aged stem cells exhibit genomic instability (Burkhalter, Rudolph, & Sperka, 2015), possibly due to a reduced capacity for repairing DNA damage induced by either endogenous or exogenous stimuli (Park, Jeon, Pyo, Kim, & Yoo, 2018; Rossi et al., 2007). However, most of this research has been conducted using DNA repair deficient mouse models or clinical samples donated by human progeria patients (Zhang et al., 2011). Whether and how physiological aging impacts genome integrity and DNA repair in adult stem cells has not been well characterized.

The major obstacles to fully understanding the link between changes in DNA repair and adult stem cell aging include the difficulty to (a) acquire adult human stem cells from a sufficient number of donors at different ages, to (b) choose appropriate types of adult stem cells for in vitro culture, and to (c) quantify the efficiency of different types of DNA repair using reliable but easy‐to‐score tools. Here, we successfully obtained 20 eyelid adipose‐derived stem cell (ADSC) lines from two groups of donors (a young group with ages ranging 17–25 years and an old group with ages ranging 50–59 years). Using these ADSCs, we systematically compared the efficiency of base excision repair (BER), nonhomologous end joining (NHEJ), and homologous recombination (HR) repair using our well‐established plasmid reactivation assay based on a GFP gene (Li et al., 2016; Mao, Jiang, Liu, Seluanov, & Gorbunova, 2009; Xu et al., 2015). Surprisingly, we found that BER efficiency but not the other two pathways for repairing DNA DSBs declines with age in human ADSCs. We also identified that XRCC1 but not other BER factors changes with age. Overexpression of XRCC1 in old ADSCs restored the decline in BER efficiency and genome integrity.

Unlike other types of adult stem cells, ADSCs can be easily obtained with minimal harm to donors and expanded in vitro with well‐established procedures (Chen et al., 2016). By analyzing the differentiation potential and surface markers, we validated the procedure for isolating ADSCs. We found that the isolated cells held the potential to differentiate into both adipocytes and osteocytes (Figure S1 A). In addition, consistent with previous reports (Locke, Windsor, & Dunbar, 2009), flow cytometry analysis indicated that the isolated ADSCs were CD13, CD29, CD44, CD90, and CD105 positive, but CD14, CD31, and CD45 negative (Figure S1 B). These results demonstrated that we successfully isolated ADSCs.

We then isolated 20 eyelid ADSC lines from donors undergoing plastic surgery (Figure S1 C). Upon isolation of the cells, we observed ~50–100 colonies of ADSCs from each cell line. When these primary ADSCs reached confluence in a 10‐cm plate, the cell numbers were ~1−2 × 10^6^. Therefore, the starting population doublings (PDs) for these cell lines were between ~13 and 15. Since DNA repair capacity may change with the increase in cumulative PDs (Mao et al., 2012; Seluanov, Mittelman, Pereira‐Smith, Wilson, & Gorbunova, 2004), we performed all the following experiments at the PD 23–25 in all cell lines (Figure S1 C). In addition, we compared the purity of the isolated ADSCs. Expression of three ADSC markers, CD13, CD44, and CD105 was tested in all isolated ADSCs at PD 23–25. We found that over 90% of the cells stained positive for the ADSC markers in all the 20 cell lines; we did not observe any significant difference between the two groups of cells (Figure S1 d–e).

**Figure 1:**
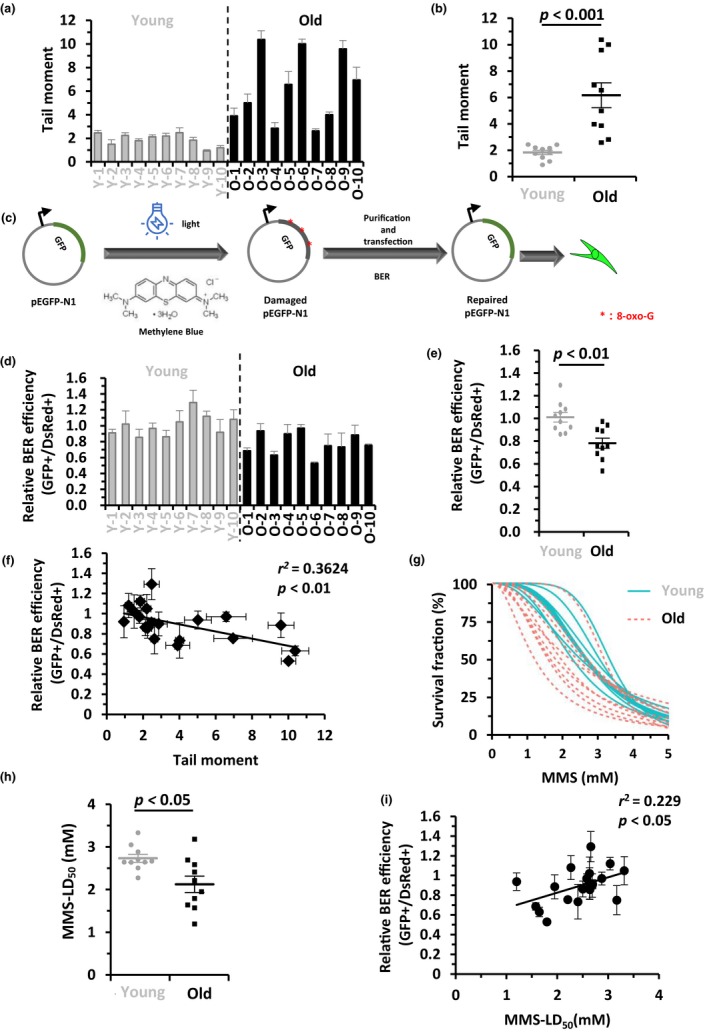
Age negatively impacts genome integrity and BER in adipose‐derived stem cells isolated from human eyelids. (a) Comparison of the genome integrity measured by alkaline comet assay between the two groups of ADSC lines. For each cell line, the tail moments of at least 50 cells were quantified using the Cometscore software. Results are presented as mean ± *SEM*. (b) Statistical analysis (Mann–Whitney *U* test) indicates that the tail moment of the ADSC lines isolated from old individuals is significantly higher than that in the young group. (c) Schematic depiction of the plasmid reactivation assay employed to measure BER efficiency. The assay is as previously described (Xu et al., 2015). The damaged pEGFP‐N1 (0.15 μg) plasmids were transfected into ADSCs together with pCMV‐DsRed (0.03 μg). Successful BER restores the GFP expression and turns the host cells green, which was quantified on FACSverse. The ratio of GFP+/DsRed + was used to calculate the BER efficiency. (d) The BER efficiencies of 20 ADSC cell lines. Results are presented as mean ± *SD*. (e) Statistical analysis (Mann–Whitney U test) of BER efficiency indicates that ADSCs from old individuals have lower BER efficiency than that in the young group. (f) BER efficiency negatively correlated with the tail moment in the 20 cell lines. (g) Young and old groups of ADSC lines were treated with increasing doses of MMS. Survival rates were calculated using DMSO treated cells as controls. The blue lines represent the young group and the red lines represent the old group. (h) Statistical analysis (Mann–Whitney U test) of the MMS‐LD_50_. (i) BER efficiency positively correlated with the MMS‐LD_50_ in the 20 cell lines

To compare the difference in genome integrity between the two groups of ADSCs, we first performed alkaline comet assays. The average tail moment of the 10 old ADSCs was 3.3‐fold higher than that of the 10 young ADSCs (Figure 1a), and the statistical analysis indicates that the tail moment of the old group of ADSCs was significantly higher than that of the young group (Figure 1b), suggesting that age has a negative impact on genome integrity.

We therefore set out to examine whether an impairment in DNA repair capacity contributes to the loss of genome integrity. Since mounting evidence indicates that the efficiency of two DNA DSB repair pathways—NHEJ and HR declines with age in somatic cells in both mice and human beings (Li et al., 2016; Vaidya et al., 2014) and that deficiency in NHEJ or HR pathway leads to a phenotype of premature aging (Espejel et al., 2004; White & Vijg, 2016), we therefore first examined if NHEJ or HR changed with age in human ADSCs using our well‐characterized, GFP‐based reporter cassettes (Li et al., 2016; Mao et al., 2009). We linearized NHEJ or HR reporter cassettes with the restriction enzyme, I‐SceI, in vitro and then transfected the digested NHEJ (0.6 µg) or HR (1.5 µg) reporter constructs together with 0.03 µg pCMV‐DsRed2, for normalizing transfection efficiency, into each exponentially proliferating ADSC line at a concentration of 8 × 10^5^ cells/ transfection. FACS analysis was performed on day 3 post‐transfection. Surprisingly, we did not observe any difference in either NHEJ efficiency (Figure S2 a–c) or HR efficiency (Figure S2 d–f) between the two groups of ADSCs. Furthermore, we analyzed the foci formation of γH2AX and 53BP1, two markers for DSBs, at different time points postionizing radiation (IR) in the young and old ADSC lines. We found that, consistent with the results of NHEJ and HR efficiency, the formation and clearance of γH2AX and 53BP1 foci did not differ between the two groups of ADSC lines (Figure S3 a–f), strongly indicating that adult stem cells retain their ability to repair DNA DSBs during aging. We next tested if age impacted BER in ADSCs using plasmid reactivation assays (Xu et al., 2015). We treated the pEGFP‐N1 plasmid with methylene blue and visible light to induce 7,8‐dihydroxy‐8‐oxoguanine and other types of single base modifications (Figure 1c) (Xu et al., 2015), followed by transfection of 0.15 µg of damaged pEGFP‐N1 together with 0.03 µg pCMV‐DsRed2. On day 3 post‐transfection, we scored the number of GFP + and DsRed + cells and employed the ratio of GFP+/DsRed + as a measure of BER efficiency (Xu et al., 2015). By comparing the BER efficiency between the two groups of ADSCs, we found that BER efficiency in the old group of ADSCs was significantly lower than that in the young group (Figure 1 d–e). To rule out the possibility that the state of quiescence or proliferation might affect the results of the plasmid reactivation assay for measuring BER efficiency, we analyzed BER efficiency in the quiescent state in two young (Y‐7 and Y‐10) and two old (O‐1 and O‐8) ADSC lines. We found that in the quiescent state, the two young ADSCs had significantly higher BER efficiency than the two old ADSCs (Figure S4 a–b), which is in agreement with the results acquired from exponentially proliferating cells.

We then examined the correlation between BER efficiency and tail moment. We observed a significant negative correlation between BER efficiency and tail moment (Figure 1f), indicating that the age‐associated decline in BER contributes to the loss of genome integrity observed in old ADSCs. Moreover, we further examined the functional consequences of this age‐associated decline in BER efficiency in ADSCs. As defects in BER sensitize cells to reactive oxygen species or alkylating agents (Bauer et al., 2011; Kondo, Takahashi, Ono, & Ohnishi, 2010), we treated cells from each ADSC line with increasing concentrations of the alkylating agent methylmethane sulfonate (MMS) and performed clonogenic assays. Afterwards, we calculated LD_50_ and found that indeed, the aged group of cells was more sensitive to MMS than the young group (Figure 1 g–h and Figure S5). We also observed a positive correlation between LD_50_ and BER efficiency (Figure 1i).

**Figure 2:**
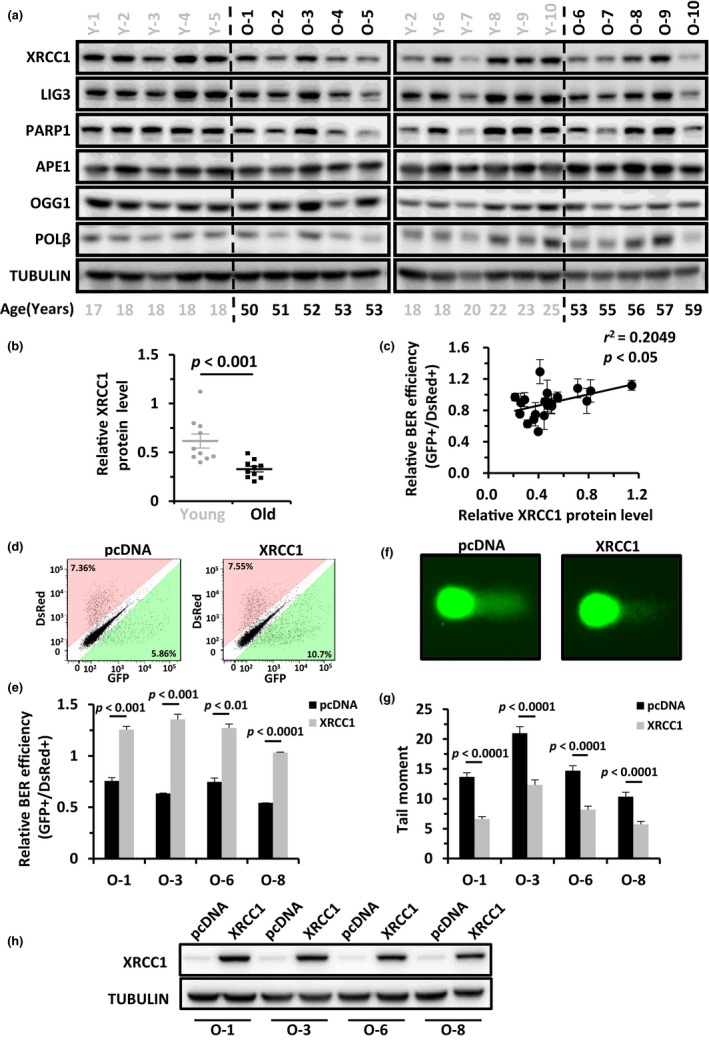
XRCC1 is a critical factor regulating age‐associated decline in BER efficiency and genome integrity. (a) Western blot analysis of the expression of all BER factors across the 20 cell lines. All cells were harvested and lysed for protein extraction on day 2 postsplitting, when cells were exponentially proliferating. (b) Mann–Whitney *U* test analysis indicates that the XRCC1 protein level in the young group of ADSCs is significantly higher than that in the old group. (c) Analysis of the correlation between XRCC1 protein level and BER efficiency. (d and e) Representative pictures (d) and statistical analyses (E) of the rescue of BER efficiency by XRCC1 overexpression in the O‐1, O‐3, O‐6, and O‐8 ADSC lines. Results are presented as mean ± *SD,* and Student's* t* test is used for statistical analysis. (f and g) Representative pictures (f) and statistical analysis (g) of the rescue of genome integrity by XRCC1 overexpression in the O‐1, O‐3, O‐6, and O‐8 ADSC lines. Results are presented as mean ± *SEM* and Student's* t* test is used for statistical analysis. (h) Western blot analysis of XRCC1 level in O‐1, O‐3, O‐6, and O‐8 ADSC lines with XRCC1 overexpressed

To understand what led to the impairment of BER in old ADSCs, we extracted proteins from the 20 ADSC lines and analyzed the protein level of multiple BER factors including XRCC1, DNA LIG3, PARP1, APE1, OGG1, POLβ, and NTHL1. We found that only XRCC1 protein level was significantly downregulated in the old group in comparison to the young group of ADSCs (Figure 2a,b), while the other six BER factors did not change at the protein level with age (Figure 2a and Figure S6 a–b). Since XRCC1 has been reported to act as a stabilization factor for LIG3 and POLβ (Caldecott et al., 1994; Fang et al., 2014; Parsons et al., 2008), we also analyzed the correlation between XRCC1 protein level and POLβ or LIG3 protein level. We found that the expression level of XRCC1 positively correlated with LIG3 expression level while no correlation was observed between XRCC1 and POLβ (Figure S7 a–b). Given the fact that XRCC1, but not POLβ or LIG3 expression, was negatively regulated by age in ADSCs, we propose that in addition to XRCC1, other factors such as Mule (Parsons et al., 2009), Iduna (Kang et al., 2011) might also participate in the regulation of POLβ or LIG3 protein levels in ADSCs.

We also performed quantitative PCR to examine if the change in XRCC1 protein level was caused by age‐associated dysregulation of transcription. However, we did not observe significant changes in mRNA level between the two groups of ADSCs (Figure S8 a). Luciferase assays were also performed to further compare the activity of the XRCC1 promoter between old and young ADSCs. No significant difference in the promoter activity between the two groups of cells was observed (Figure S8 b–c). These results indicate that the age‐associated decline of XRCC1 protein level is probably caused by other post‐transcriptional or post‐translational mechanisms. Also, we observed a positive correlation between XRCC1 protein level and BER efficiency in the 20 ADSC lines (Figure 2c), further highlighting the age‐dependent relationship between XRCC1 and BER efficiency.

We then examined if overexpressing XRCC1 in four of the old ADSC lines and two of the young ADSC lines could enhance BER. We found that overexpressing XRCC1 could stimulate the BER efficiency in all six of the ADSC lines (Figure 2d,e and Figure S9). Overexpression of XRCC1 rescued the decline of genome integrity in the old ADSC lines (Figure 2 f–h). In addition, we found that XRCC1 stimulates BER in a dose‐dependent manner (Figure S10). Moreover, depleting XRCC1 in young ADSCs significantly inhibited BER efficiency and resulted in a loss of genome integrity assessed by comet assay (Figure S11 a–c).

To further test whether age impacts the functions of ADSCs, we examined the osteogenic differentiation potential of ADSCs with Alizarin Red S staining assay. We found that, in agreement with previous reports (Liu et al., 2017), the differentiation potential was impaired in the old groups of ADSCs in comparison to the young cells (Figure S12 a–b). However, we did not observe any significant correlation between the differentiation potential and BER efficiency or XRCC1 expression (Figure S12 c–d). A previous report indicates that during the process of adipogenic differentiation of ADSCs the capacity to repair H_2_O_2_‐induced damage declines (Valverde et al., 2018), indicating that BER pathway might play a role in regulating differentiation potential of ADSCs. Therefore, we infected two of the old ADSC lines with lentivirus bearing an XRCC1 expression vector and examined their differentiation potential. Surprisingly, although the effect was relatively mild (~ 20%), XRCC1 overexpression significantly improved the potential of adipogenic differentiation (Figure S13 a–c). Taken together, these results indicate that improving XRCC1 expression not only stabilizes genomes but also helps promote the differentiation potential of ADSCs.

Here, for the first time, with a collection of ADSC lines isolated from different donors, we demonstrated that age has a negative effect on the BER pathway, but not either of the major DSB repair pathways. Our data also indicate that targeting XRCC1 is a promising method to stabilize genomes and improve the functionality of ADSCs. However, whether improving genome integrity by activating the BER pathway directly improves ADSC functionality and what causes the age‐related decline in XRCC1 protein level are questions that remain to be determined.

## CONFLICT OF INTERESTS

None declared.

## AUTHOR CONTRIBUTIONS

Haiping Zhang and Bailian Cai were involved in ADSC isolation, cell culture, cell differentiation, DNA repair analysis, comet assay, manuscript writing, data analysis, and interpretation. Anke Geng and Sheng Li were involved in data analysis and interpretation, and collection and assembly of data. Huanyin Tang was involved in statistical analysis and graphing. Wenjun Zhang was involved in collection of clinical samples. Ying Jiang assisted with the provision of study material and collection of data. Rong Tan, Xiaoping Wan, and Zhiyong Mao were involved in the conception and design, data interpretation, manuscript writing, and final approval of manuscript.
