# Supplementary material for: Base excision repair but not DNA double‐strand break repair is impaired in aged human adipose‐derived stem cells
Source: Aging Cell. 2019 Nov 29;19(2):e13062. doi: 10.1111/acel.13062 (PMC6996963; doi:10.1111/acel.13062)
Supplement: Supplementary file 1 [file ACEL-19-e13062-s001.docx]

## SUPPORTING INFORMATION

- **Experimental procedures**
- **Supplementary Figures (Figure S1-S13)**

**EXPERIMENTAL PROCEDURES**

**Antibodies, reagents and siRNA**

The information of antibodies used in FACS and Western blot experiments are as follows:γH2AX (Cat. # 9718S, Cell Signaling, Boston, USA), 53BP1 (Cat. # ab36823, Abcam, Cambridge, MA, USA), XRCC1 (Cat. # ab134056, Abcam, Cambridge, MA, USA), PARP1 (Cat. #A3121, Abclonal, Boston, USA), LIG3 (Cat. # A1887, Abclonal, Boston, USA), OGG1 (Cat. # A2268, Abclonal, Boston, USA), APE1 (Cat. #A1117, Abclonal, Boston, USA), Pol β (Cat. #A1681, Abclonal, Boston, USA), NTHL1 (Cat. # ab70726, Abcam, Cambridge, MA, USA), TUBULIN (Cat. # AP0064, Bioworld, USA) CD13 (Cat. # ab7417, Abcam, Cambridge, MA,USA)，CD14-FITC (Cat. # ab28061, Abcam, Cambridge, MA,USA)，CD29 (Cat. # ab30394, Abcam, Cambridge, MA, USA)，CD31 (Cat. # ab9498, Abcam, Cambridge, MA,USA)，CD44 (Cat. # ab119863, Abcam, Cambridge, MA,USA)，CD45 (Cat. # ab10558, Abcam, Cambridge, MA,USA)，CD90-FITC (Cat. # ab11155, Abcam, Cambridge, MA,USA)，CD105-FITC (Cat. # ab53318, Abcam, Cambridge, MA,USA).The information of reagents used in this article is as follows: biotin (Cat. # B4639, Sigma, Darmstadt, Germany), D-pantothenate (Cat. # P5155, Sigma, Darmstadt, Germany), dexamethasone (Cat. # D4902, Sigma, Darmstadt, Germany), insulin (Cat. # 91077C, Sigma, Darmstadt, Germany), rosiglitazone (Cat. # R2408, Sigma, Darmstadt, Germany), methylisobutylxanthine (Cat. # I7018, Sigma, Darmstadt, Germany), ascorbate-2-phosphate (Cat. # A8960, Sigma, Darmstadt, Germany),β-glycerophosphate (Cat. # G5422, Sigma, Darmstadt, Germany), Oil red O (Cat. # O9755, Sigma, Darmstadt, Germany), Alizarin red S (Cat. # A5533, Sigma, Darmstadt, Germany). The sequence of siRNA against XRCC1 is as follows:

5’-CCGAUGGAUCUACAGUUGCAA TT-3’.

**Isolation and culture of human adipose-derived stem cells**

All human samples used in this study were obtained with the consent of their donors and were in conformity with the Ethic Committees of Tongji University and Changzheng Hospital (2015yxy111). The donors were in good health. The eyelid adipose tissues were acquired from plastic surgery and kept in 4 ºC PBS. The ADSC isolation assay was initiated within 24 hours post surgery. The isolation procedure was performed as previously reported (Estes et al., 2010) with some small modifications. Briefly, adipose tissues were washed once or twice in PBS to remove blood cells, then the tissue was immediately minced into tiny pieces by sterile scissors and digested in the collagenase type I (PBS with 10 mg/ml BSA, 1 mg/ml collagenase type I and 2 mM CaCl_2_) for 90 minutes at 37 ℃. After being centrifuged at 1500 rpm for 5 minutes, the precipitates were resuspended in DMEM medium supplemented with 10% FBS and 1% penicillin/streptomycin and plated into 10-cm dishes. Cells were maintained in Heracell 240i incubators (Thermo Fisher, Waltham, MA, USA) with 5% CO_2_ and 3% O_2_ at 37 °C. All cultured cells were counted on a CounterStar machine and the PD number was calculated as previously reported (Greenwood et al., 2004).

**Adipogenic differentiation**

The ADSCs were seeded in 24 or 12 wells plates at 25,000 or 50,000 cells per well. Once cells reached confluence, the stromal medium was replaced with the differentiation medium (Yu et al., 2011) which contains DMEM medium (Cat. # 11995-065, Gibco, Carlsbad, CA, USA), 3% FBS (Cat. # 16000-044, Gibco, Carlsbad, CA, USA), 1% penicillin/streptomycin (Cat. # 15140-122, Gibco, Carlsbad, CA, USA), 33 μM biotin, 17 μM D-pantothenate, 1 μM dexamethasone, 0.1 μM insulin, 5 μM rosiglitazone and 500 μM methylisobutylxanthine. On day three post the medium change, the differentiation medium was replaced with the adipocyte maintenance medium (DMEM medium, 3% FBS, 1% penicillin/streptomycin, 33 μM biotin, 17 μM D-pantothenate, 1 μM dexamethasone, 0.1 μM insulin). The maintenance medium was regularly changed every 3 days for 2 weeks.

**Oil Red O staining and quantification**

Oil Red O staining solution was prepared as previous reported (Yu et al., 2011). Briefly，to prepare the Oil Red O staining stock solution, 0.35 g Oil Red O (Cat. # O9755, Sigma, Darmstadt, Germany) was dissolved in 100 ml isopropanol and filtered through a 0.2-μm filter. Then, the staining solution was prepared by mixing 60 ml stock solution with 40 ml sterile distilled water and was filtered through a 0.2-μm filter again before use.

The differentiated cells were rinsed 3 times with cold PBS and fixed with 10% formaldehyde for 60 minutes at room temperature or overnight at 4 ℃. Next, fixed cells were incubated with the prepared Oil Red O staining solution for 15 minutes, followed by three PBS washes. Pictures were taken on an inverted microscope (Nikon, Tokyo, Japan).

For quantification, Oil Red O stained cells were eluted by 100% isopropanol (0.2 ml/well for 12-well plate and 0.1 ml/well for 24-well plate). A blank well without cells was treated by the same method as a control. The absorbance (OD540) was determined at 540 nm on a Multiscan Spectrum (SpectraMax® M5, Molecular Devices, California, USA). Results were presented as the OD values of sample wells with blank wells subtracted.

**Osteogenic differentiation**

Once ADSCs reached confluence, the osteogenic differentiation medium (DMEM medium, 10% FBS, 1% penicillin/streptomycin, 0.1 mM dexamethasone, 50 mM ascorbate-2-phosphate (Cat. # A8960, Sigma, Darmstadt, Germany), and 10 mM β-glycerophosphate (Cat. # G5422, Sigma, Darmstadt, Germany) were added to the plates to replace the stromal medium. The medium was changed every 2 days for 3 weeks.

**Alizarin Red S staining and quantification**

The procedure for Alizarin red S staining is similar to that of the Oil red O staining. The only different steps are the preparation of Alizarin Red S staining solution. Before the staining step, 0.1 g Alizarin Red S (Cat. # A5533, Sigma, Darmstadt, Germany) was dissolved in Tris solution (0.01 g/ml, pH 8.3). The solution was filtered through a 0.2-μm filter before use. The staining step was the same as Oil Red O staining. For quantification, Alizarin Red S stained cells were eluted by release buffer (10% acetic acid, 20% methanol, 70% sterile distilled water) (0.2 ml/well for 12-well plate and 0.1 ml/well for 24-well plate). The absorbance was determined at 450 nm.

**Flow cytometry for** **immunophenotyping**

CD13，CD14-FITC，CD29，CD31，CD44，CD45，CD90-FITC，CD105-FITC antibodies were used for immunophenotyping. Exponentially proliferating ADSCs at PD 20 were harvested and re-suspended in cold PBS containing 2% BSA, followed by shaking at room temperature for 1 hour. Then, the ADSCs were incubated in cold PBS containing the antibodies of surface markers at 4℃ overnight. Subsequently, cells were centrifuged at 1500 rpm for 5 mins, and then washed three times with cold PBS. For those primary antibodies conjugated with FITC, the cells were resuspended and analyzed on FACSverse (BD Biosciences, San Jose, CA, USA). For those primary antibodies not conjugated with FITC, secondary antibodies were incubated with ADSCs at 25 ℃ for 1 hour and washed with cold PBS for three times. Finally, cells were resuspended in 200 µl cold PBS and analyzed on FACSverse (BD Biosciences, San Jose, CA, USA).

**Analysis of efficiency of DNA damage repair**

Cells were pre-seeded at 5 × 10^5^ cells/plate in 10 cm plates. On day 2 post splitting, I-SceI linearized NHEJ (0.6 μg) construct or HR (1.5 μg) construct or methylene blue + vision light treated pEGFP-N1 vector (0.15 μg) together with pCMV-DsRed (0.03 μg) were electroporated into ADSCs with EX147 program on a Lonza 4D machine (Lonza, Cologne, Germany). On day 3 post transfection, cells were harvested for FACS analysis on FACSverse. The ratio of GFP+/DsRed+ was used to calculate the DNA repair efficiency. The data were analyzed by FlowJo (Ashland, OR, USA).

**Comet assay**

Exponentially proliferating ADSCs at PD 23 - 25 were harvested and lysed for alkaline comet assay with a kit from Trevigen (Cat. #: 4250-050-K, Trevigen, Gaithersburg, MD, USA) according to the manufacturer’s instructions.

**Clonogenic assay**

Cells were seeded in 6-well plates at a density of 200 cells/well. After 24 hours, the cells were treated with MMS at a concentration of 0 mM, 1 mM, 2 mM, 3 mM, 4 mM and 5 mM for 30 mins and replaced with fresh medium. After a 2-week culture in the incubator, the formed colonies were stained with coomassie reagent (methanol: acetic acid: Coomassie: H_2_O= 50: 10: 0.25: 40) for 3 hours at room temperature. Afterwards, they were washed with ddH_2_O and colonies with at least 50 cells were counted. Survival rate was calculated as the relative plating efficiencies of the MMS treated to controls.

**Immunofluorescence assay**

Cells were seeded on coverslips in 12-well plates at a density of 2 × 10^4^ cells. 48 hours later, the cells were treated with X-ray at a dose of 2 Gy and then cultured for 2 hours or 16 hours before immunostaining experiments were performed. Next, cells were washed with cold PBS and then fixed with 4% paraformaldehyde (PFA) for 15 min at room temperature. Fixed cells were washed once with PBS and permeablized with 0.25% Triton X-100 (Cat. #X100, Sigma, Darmstadt, Germany) for 10 min at room temperature. Cells then were washed three times with cold PBS and blocked by 2% bovine serum albumin (BSA) and followed by incubation with primary antibodies for 4 hours and secondary antibody for 1 hour at room temperature. After being stained with DAPI, the cells were covered and pictures were taken with a laser scanning confocal microscope (TCS SP8; Leica, Wetzlar, Germany).

**Luciferase assay**

The XRCC1 promoter was amplified from the genomic DNA of a young ADSC line, with the primers:

5’-GGTACCGAGCTCTTACGCGTGGACGCAGAACCCTTCTCTTTTGGC-3’ and 5’-AGCTTACTTAGATCGCAGATCTCGAGGAGTCCTGGCTGCTGCAGGAC-3’. Then the ~ 1 kb DNA segment was cloned into pGL3-basic plasmid and verified by sequencing. The 20 ADSC cells were seeded at a density of 5 × 10^5^ cells in 10-cm plates and cultured for 48 hours. Then the luciferase reporters (6 μg) together with Renilla vector (6 ng) were electroporated into ADSCs with EX147 program on a Lonza 4D machine (Lonza, Cologne, Germany). At 48 hours post transfections, cells were harvested, and luciferase activity was measured with a dual luciferase reporter system (Cat. #E1910, Promega, Madison, WI, USA) and a GloMax Luminometer (Cat. #E5311, Promega, Madison, WI, USA).

**REFERENCES**

Estes, B. T., Diekman, B. O., Gimble, J. M., & Guilak, F. (2010). Isolation of adipose-derived stem cells and their induction to a chondrogenic phenotype. *Nat Protoc, 5*(7), 1294-1311. doi:10.1038/nprot.2010.81

Greenwood, S. K., Hill, R. B., Sun, J. T., Armstrong, M. J., Johnson, T. E., Gara, J. P., & Galloway, S. M. (2004). Population doubling: a simple and more accurate estimation of cell growth suppression in the in vitro assay for chromosomal aberrations that reduces irrelevant positive results. *Environ Mol Mutagen, 43*(1), 36-44. doi:10.1002/em.10207

Li, Z., Zhang, W., Chen, Y., Guo, W., Zhang, J., Tang, H., . . . Mao, Z. (2016). Impaired DNA double-strand break repair contributes to the age-associated rise of genomic instability in humans. *Cell Death Differ, 23*(11), 1765-1777. doi:10.1038/cdd.2016.65

Mao, Z., Jiang, Y., Liu, X., Seluanov, A., & Gorbunova, V. (2009). DNA repair by homologous recombination, but not by nonhomologous end joining, is elevated in breast cancer cells. *Neoplasia, 11*(7), 683-691.

Yu, G., Wu, X., Kilroy, G., Halvorsen, Y. D., Gimble, J. M., & Floyd, Z. E. (2011). Isolation of murine adipose-derived stem cells. *Methods Mol Biol, 702*, 29-36. doi:10.1007/978-1-61737-960-4_3

**SUPPLEMENTARY FIGURES**


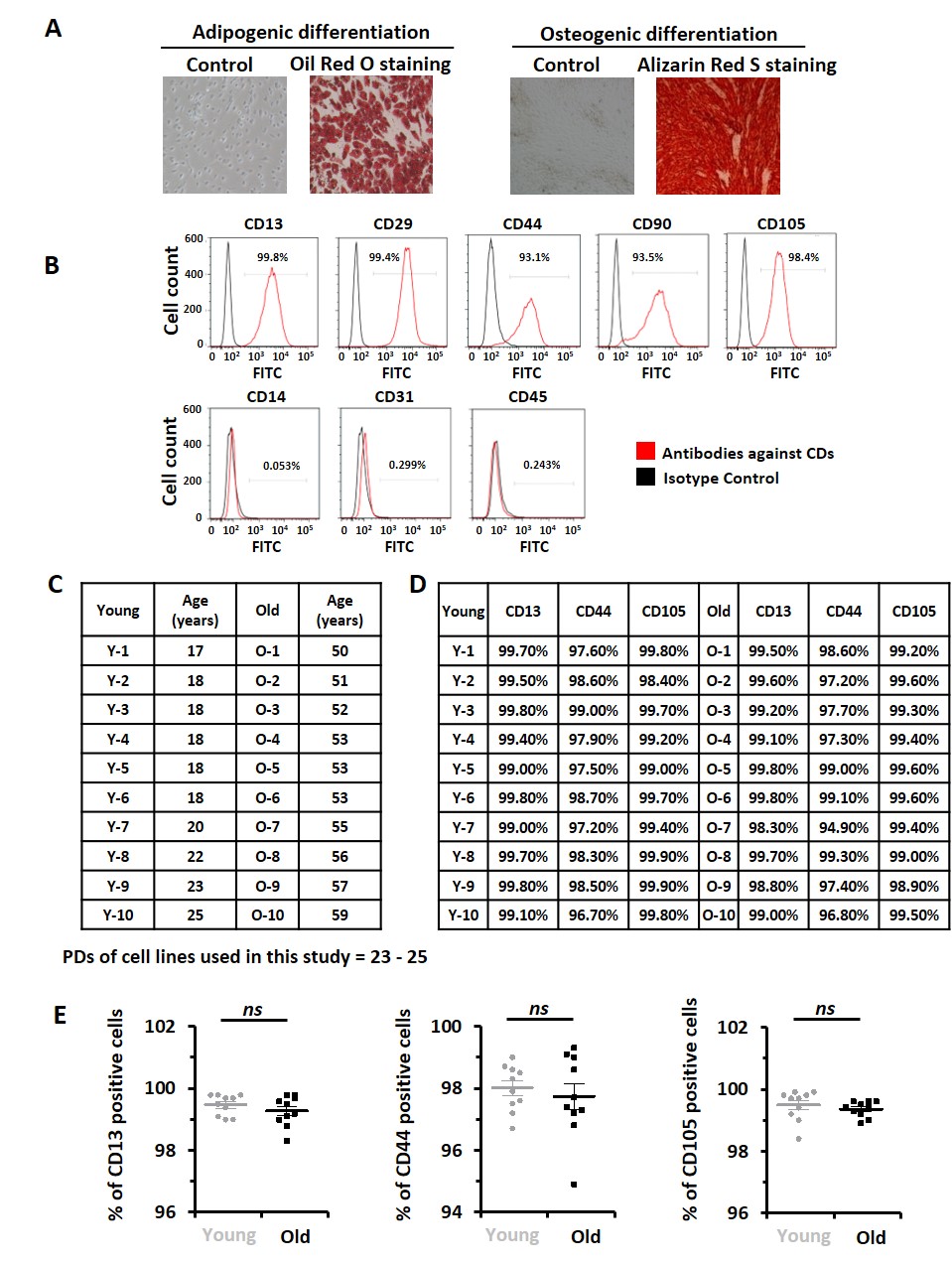


**Figure S1**

**FIGURE S1. Isolation and validation of ADSC lines.** (A) Representative pictures of adipogenic and osteogenic differentiation of ADSC lines. (B) The expression profile analysis of surface markers of ADSCs by flow cytometry analysis. (C) The age of 20 donors and the PDs of cell lines used in this study. PDs：Population doublings. (D)The expression profile analysis of three positive surface markers of the 20 ADSC lines. All cells were analyzed at the PDs of 23-25. (E) The comparison of purity between the two groups of ADSCs. Mann-Whitney U Test was performed to compare the differences. *ns*: not significant.

**
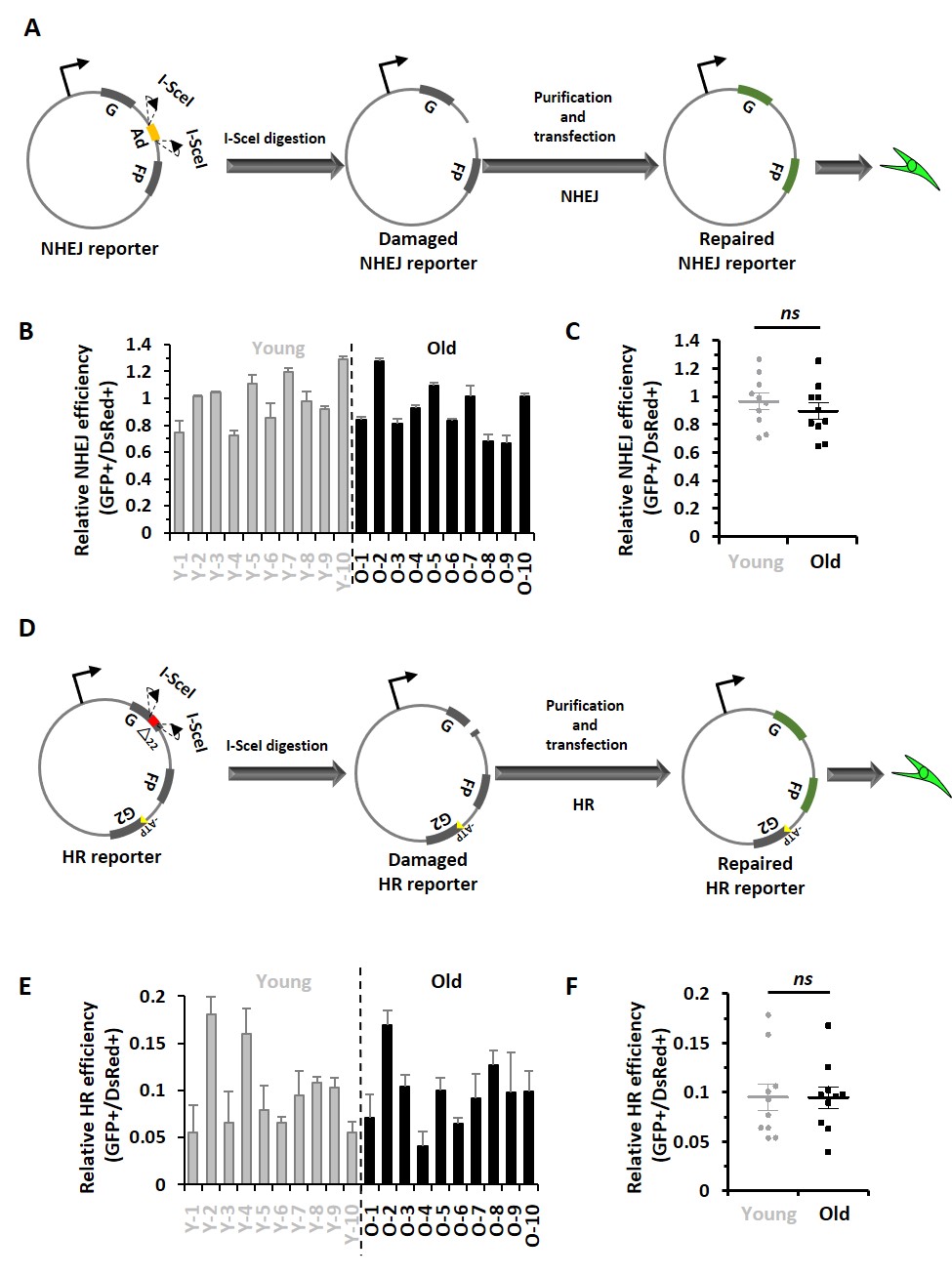
**

**Figure S2**

**FIGURE S2. The efficiencies of NHEJ and HR show no difference between young and old ADSCs.** (A)(D) are schematic depictions of the plasmid reactivation assay used to analyze NHEJ(A) and HR(D) efficiency. (B)(E) are NHEJ (B) and HR (E) efficiency of the two groups, respectively. Results are presented as mean ± *SD*. (C)(F) are statistical analysis of NHEJ (C) and HR (F) efficiency between two groups, respectively. Mann-Whitney U Test was performed to compare the difference. *ns*: not significant.


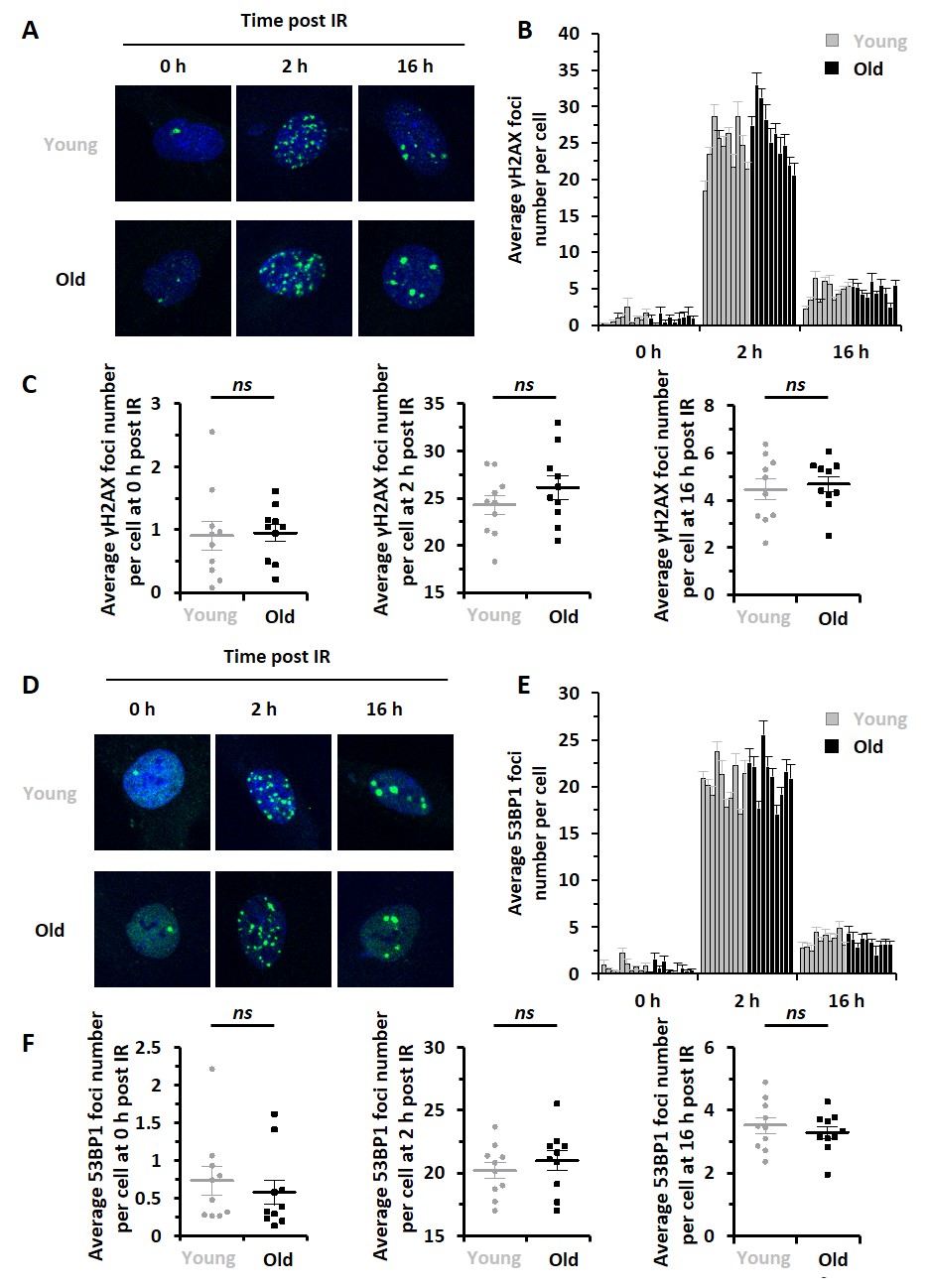


**Figure S3**

**FIGURE S3. Average foci number of γH2AX and 53BP1 in the 20 ADSC lines.** (A)(D) Representative pictures of γH2AX and 53BP1 foci at different time points post IR (X-ray, 2 Gy) in one young and one old cell line, respectively. (B)(E) Quantification of γH2AX and 53BP1 foci numbers at different time points post IR in the 20 cell lines. At least 50 cells were counted for each time point. Results are presented as mean ± *SD*. (C)(F) Statistic analysis of γH2AX foci (C) and 53BP1 foci (F) between the young and old groups at different time points post IR. Mann-Whitney U Test was performed to compare the difference. *ns*: not significant.


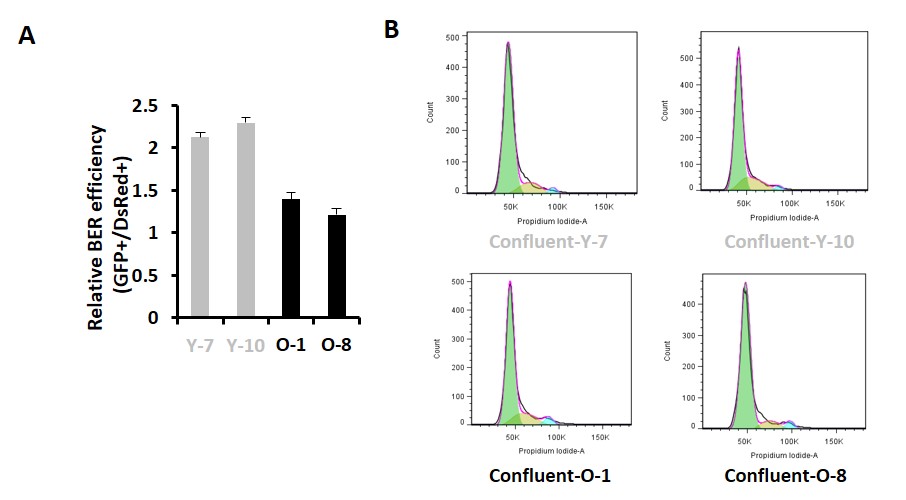


**Figure S4**

**FIGURE S4. The BER efficiency in two young ADSCs is significantly higher than that in two old ADSCs in the quiescent state.** (A) The BER efficiency of two young and two old ADSC lines in the quiescent state. Results are presented as mean ± *SD.* (B) Cell cycle distribution of two young and two old ADSC cell lines in the quiescent state.


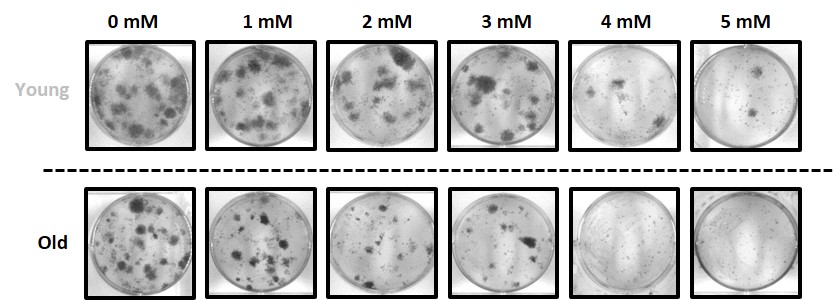


**Figure S5**

**FIGURE S5. Representative pictures from clonogenic assays.** Cells were treated with methylmethane sulfonate (MMS) at a concentration of 0 mM, 1 mM, 2 mM, 3 mM, 4 mM and 5 mM in one young and old cell line, respectively. Then clonogenic assay was performed and pictures were taken.


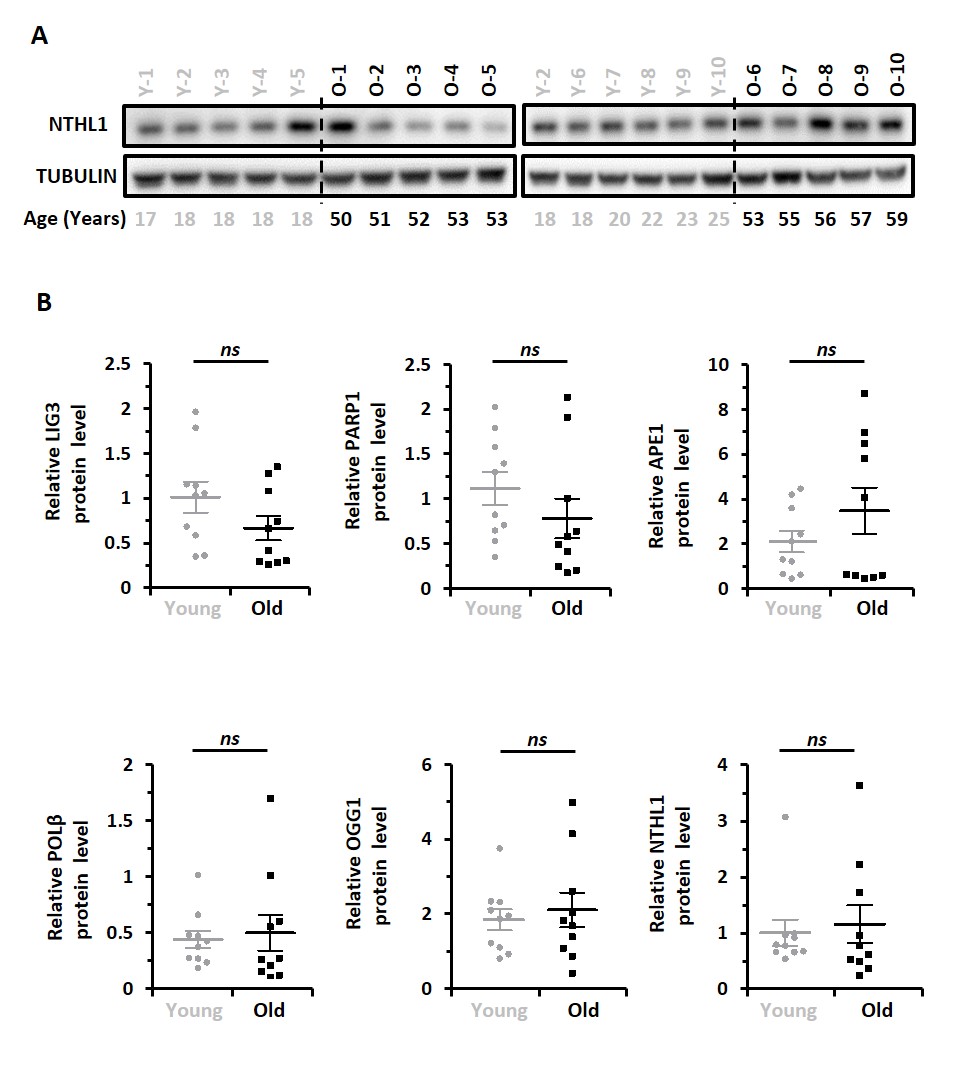


**Figure S6**

**FIGURE S6. The comparison of protein levels of LIG3, PARP1, APE1, POLβ, OGG1 and NTHL1 between young and old ADSC groups.** (A) Western blot analysis of the expression of NTHL1 in the 20 ADSC lines. (B) Mann-Whitney U test analysis indicates that the protein levels of LIG3, PARP1, APE1, POLβ, OGG1 and NHTL1 show no difference between the young and old ADSC groups. *ns*: not significant.

**
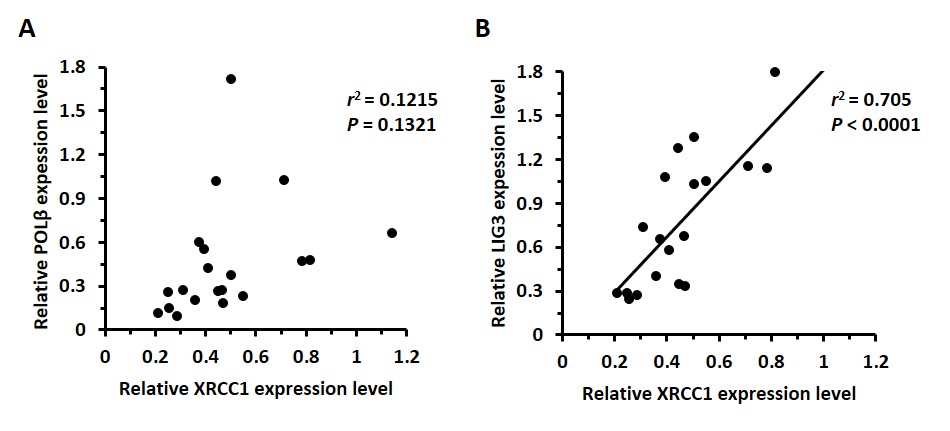
**

**Figure S7**

**FIGURE S7. Correlation of expression levels of XRCC1 and other two BER factor POLβ and LIG3 respectively.** (A) Analysis of the correlation between XRCC1 protein level and POLβ protein level. (B) Analysis of the correlation between XRCC1 protein level and LIG3 protein level.


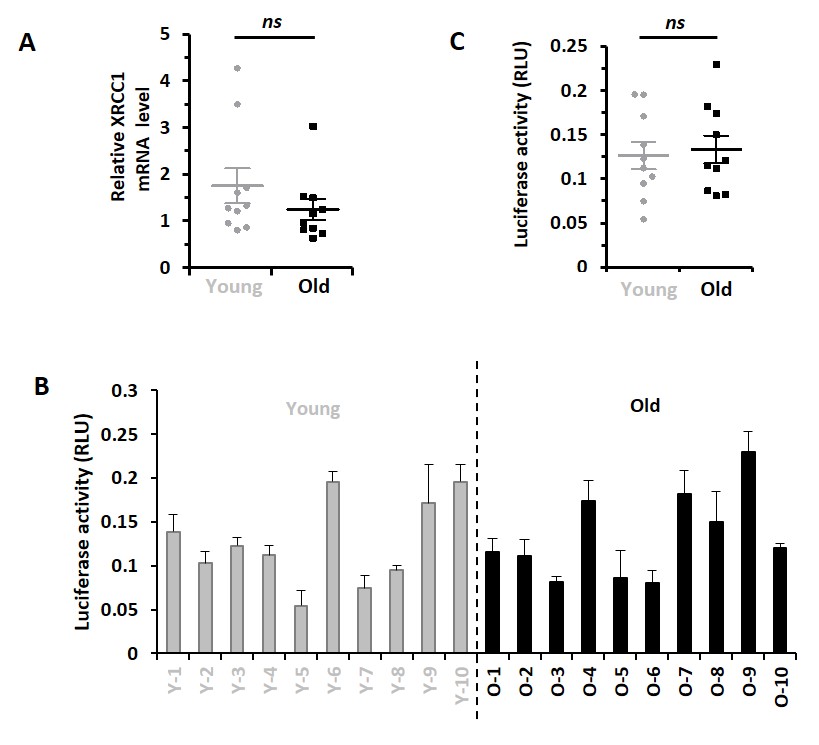


**Figure S8**

**FIGURE S8. XRCC1 mRNA level and promoter activity exhibit no difference between the young and old groups of ADSCs.** (A) Mann-Whitney U test analysis of relative XRCC1 mRNA level indicates that the XRCC1 mRNA has no significant difference between the two groups. All cells were harvested and lysed for RNA extraction and qRT-PCR on day 2 post splitting, when cells were exponentially proliferating. *ns*: not significant. (B) The luciferase assay indicates that the promoter activity of XRCC1 did not change in old ADSCs in comparison to that in young ADSCs. Results are presented as mean ± *SD*. (C) Mann-Whitney U test analysis of promoter activity. *ns:* no significant.

**
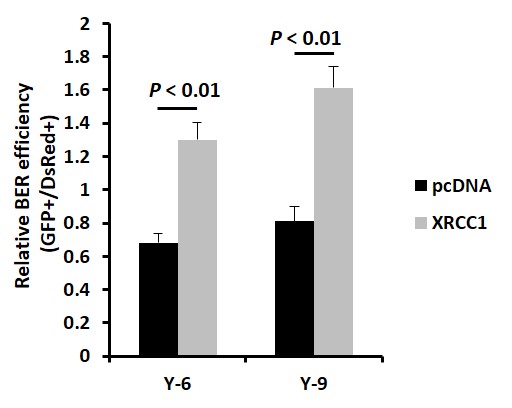
**

**Figure S9**

**FIGURE S9. BER efficiency is stimulated by XRCC1 overexpression in two young ADSCs.** Results are presented as mean ± *SD* and Student *t* Test is used for statistical analysis.


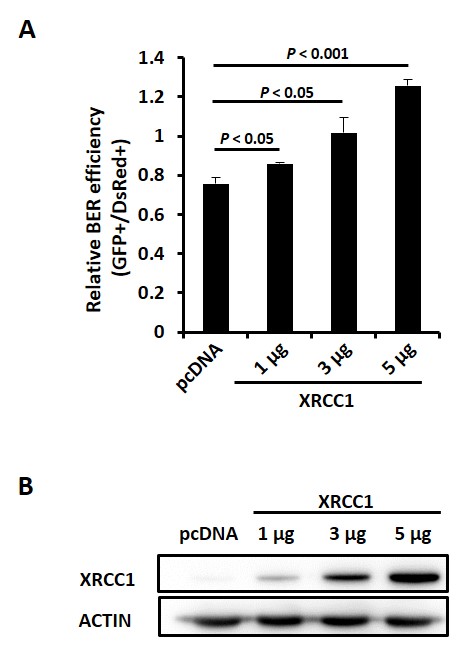


**Figure S10**

**FIGURE S10. XRCC1 stimulates BER in a dose dependent manner.** (A) BER efficiency is enhanced in a dose dependent manner in one old ADSC line (O-1) with increasing amounts of XRCC1 transfected. Results are presented as mean ± *SD* and Student *t* Test is used for statistical analysis. (B) Western blot analysis of XRCC1 expression in the ADSCs with different amounts of XRCC1 vectors electroporated.


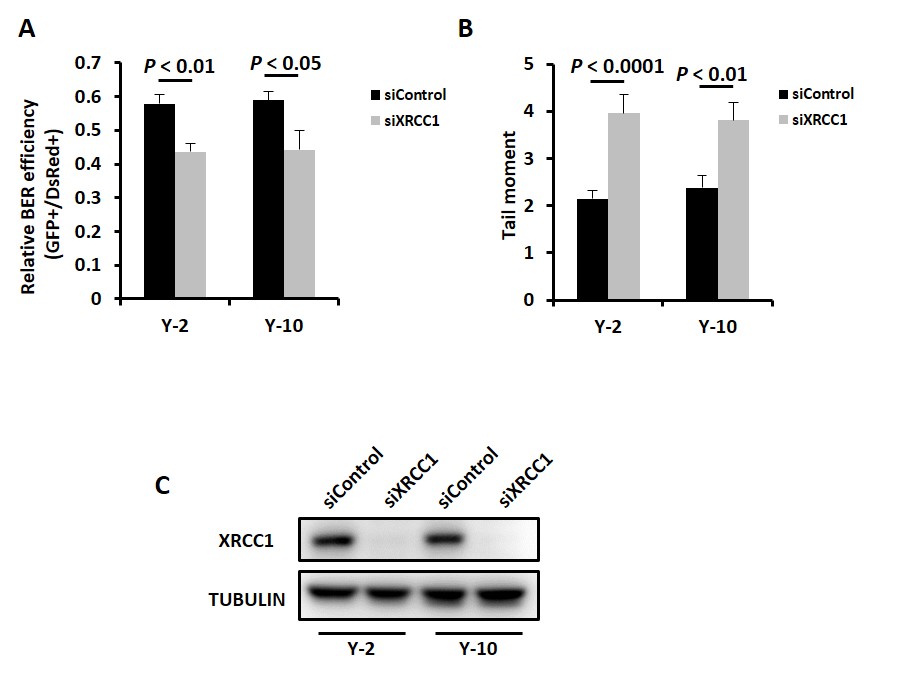


**Figure S11**

**FIGURE S11. Depleting XRCC1 negatively affects BER and genome integrity in two young ADSC lines.** (A)(B) BER efficiency (A) and tail moment (B) in two young ADSC lines with XRCC1 depleted using siRNA. Results are presented as mean ± *SD* and Student *t* Test is used for statistical analysis. (C) Western blot analysis of XRCC1 protein levels in Y-2 and Y-10 ADSC lines.


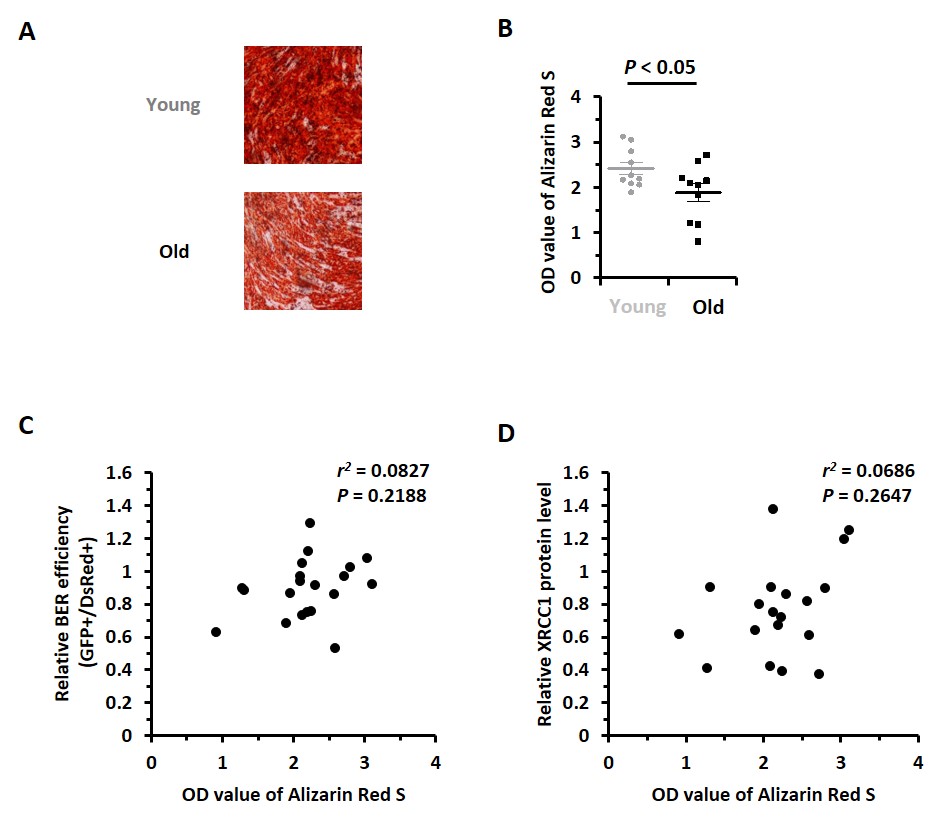


**Figure S12**

**FIGURE S12. The osteogenic differentiation potential is impaired in the old group of ADSCs.** (A) Representative pictures and (B) Statistic analysis of osteogenic differentiation assayed by OD value of Alizarin Red S staining in the two groups of ADSCs. Mann-Whitney U Test was employed for statistical analysis. (C) Analysis of the correlation between OD value of Alizarin Red S staining and BER efficiency in the 20 cell lines. (D) Analysis of the correlation between OD value of Alizarin Red S staining and XRCC1 expression in the 20 cell lines.


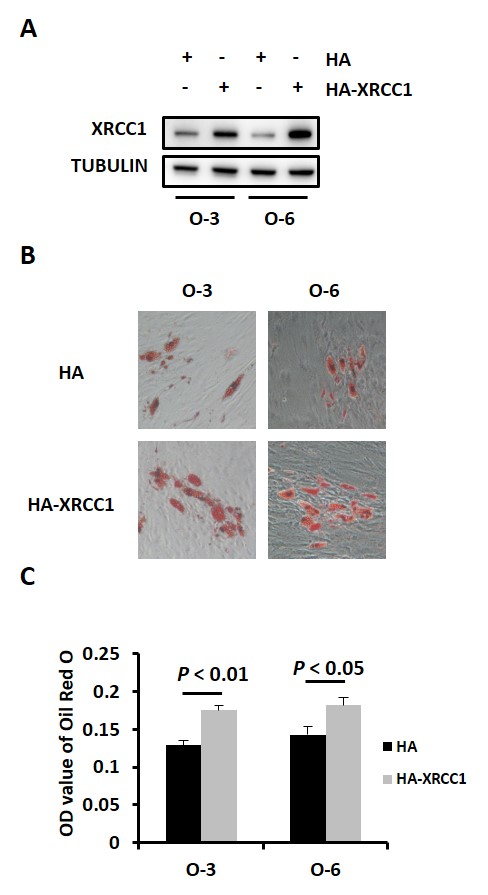


**Figure S13**

**FIGURE S13. XRCC1 promotes the adipogenic differentiation potential of ADSCs.** (A) Western blot analysis of XRCC1 protein levels in two old ADSC lines infected with lentiviruses bearing a HA control vector or a HA-XRCC1 expression vector. (B) Representative pictures of adipogenic differentiation of two old ADSC lines with XRCC1 overexpressed. (C) Comparison of the adipogenic differentiation efficiency assayed by OD value of Oil Red O in the two old ADSC lines. Results are presented as mean ± *SD* and Student *t* Test is used for statistical analysis.
